# Clinical characteristics and outcomes of infection with human T-lymphotropic virus in a non-endemic area: a single institution study

**DOI:** 10.3389/fmicb.2023.1187697

**Published:** 2023-06-22

**Authors:** Margery Gang, Feng Gao, Sneha Poondru, Theodore Thomas, Lee Ratner

**Affiliations:** ^1^Division of Oncology, Department of Medicine, Washington University School of Medicine, St. Louis, MO, United States; ^2^Department of Surgery at Barnes-Jewish Hospital and Alvin Siteman Cancer Center, Cancer Center Biostatistics Core, Division of Public Health Sciences, St. Louis, MO, United States; ^3^St Louis Veterans Health Administration Medical Center Research Service, St. Louis, MO, United States

**Keywords:** HTLV, epidemiology, non-endemic, hepatitis C, United States

## Abstract

**Introduction:**

Understanding of human T-lymphotropic virus (HTLV) remains largely based on epidemiologic and clinical data from endemic areas. Globalization has resulted in migration of persons living with HTLV (PLHTLV) from endemic to non-endemic areas, and a rise of HTLV infection in the United States. Yet, due to the historical rarity of this disease, affected patients are often under- and mis-diagnosed. Thus, we sought to characterize the epidemiology, clinical features, comorbidities, and survival of HTLV-1- or HTLV-2-positive individuals identified in a non-endemic area.

**Methods:**

Our study was a single institution, retrospective case–control analysis of HTLV-1 or HTLV-2 patients between 1998 and 2020. We utilized two HTLV-negative controls, matched for age, sex, and ethnicity, for each HTLV-positive case. We evaluated associations between HTLV infection and several hematologic, neurologic, infectious, and rheumatologic covariates. Finally, clinical factors predictive of overall survival (OS) were assessed.

**Results:**

We identified 38 cases of HTLV infection, of whom 23 were HTLV-1 and 15 were HTLV-2 positive. The majority (~54%) of patients in our control group received HTLV testing for transplant evaluation, compared to ~24% of HTLV-seropositive patients. Co-morbidities associated with HTLV, hepatitis C seropositivity were higher in HTLV-seropositive patients compared to controls (OR 10.7, 95% CI = 3.2–59.0, *p* < 0.001). Hepatitis C and HTLV co-infection resulted in decreased OS, compared to no infection, hepatitis C infection alone, or HTLV infection alone. Patients with any cancer diagnosis and HTLV infection had worse OS compared to patients with cancer or HTLV alone. HTLV-1 positive patients had lower median OS compared to HTLV-2 patients (47.7 months vs. 77.4 months). In univariate analysis, the hazard for 1-year all-cause mortality was increased among patients with HTLV-seropositivity, adult T-cell leukemia, acute myelogenous leukemia, and hepatitis C infection. When corrected, multivariate analysis showed that HTLV seropositivity was no longer associated with 1 year all-cause mortality; however association with AML and hepatitis C infection remained significant.

**Conclusion:**

HTLV-seropositivity was not associated with increased 1 year mortality in multivariate analysis. However, our study is limited by our small patient sample size, as well as the biased patient control population due to selection factors for HTLV testing.

## Introduction

The first human oncogenic retrovirus discovered, human T-lymphotropic virus type 1 (HTLV-1) affects around 10 to 20 million individuals worldwide (Gessain and Cassar, 2012). HTLV-1 is spread through breastfeeding, sexual intercourse, blood transfusions, and needle sharing. With foci of endemicity in Japan, the Caribbean, and Central and South America, HTLV-1 preferentially infects CD4+ T-cells and has lifelong latency. The vast majority of infected individuals remain asymptomatic carriers, however persons living with HTLV (PLHTLV) can undergo transformation into adult T-cell leukemia (ATL), an aggressive mature T-cell malignancy, or can develop HTLV-1 associated myelopathy (HAM), a progressive chronic central nervous system disease (Murphy et al., 1999; Gonçalves et al., 2008; Yamano and Sato, 2012). Rarely, HTLV-1 presents with inflammatory arthropathy, uveitis, pneumonitis, myositis, or thyroiditis (Gonçalves et al., 2010). The subsequent immunosuppression caused by dysfunctional HTLV-1 infected T-cells also allows for opportunistic parasitic (i.e., Strongyloides) and fungal infections (Goncalves et al., 2008).

Despite sharing considerable structural homology and transmission routes with HTLV-1, human T-lymphotropic virus type 2 (HTLV-2) has a distinct geographic spread, with endemic regions in indigenous populations in the Americas and cohorts of intravenous drug users in the United States and Europe (Khabbaz et al., 1992; Hall et al., 1996; Paiva and Casseb, 2015; Martinez et al., 2019; Ishak et al., 2020; Abreu et al., 2022). HTLV-2 has not been causally linked to hematologic disease, although cases of neurological pathologies have been proposed (Hjelle et al., 1992; Jacobson et al., 1993).

Much of our current knowledge of HTLV is based on research in endemic areas. A prospective cohort study in Nagasaki identified 1997 HTLV-1+ patients and found that carrier status was associated with increased risk of all-cause mortality and development of non-neoplastic diseases and heart disease (Iwata et al., 1994). Another retrospective study in Israel found high incidences of malignancies among PLHTLV, including ATL, mycosis fungoides, cervical carcinoma, and transitional cell carcinoma (Stienlauf et al., 2013). Few large studies have been conducted in the United States. The most notable epidemiological study by Murphy et al. screened 1.7 million blood bank donors in 1999 and identified 156 HTLV-1+ and 384 HTLV-2+ PLHTLV in the United States. In this study, HTLV-1 was associated with black ethnicity and HCV seropositivity, and HTLV-2 infection was associated with a distinct birth cohort (age 30–49 years), female sex, black and Hispanic ethnicity, and HCV seropositivity (Murphy et al., 1999; Chang et al., 2014).

In recent years there has been an increased incidence of HTLV in the United States. However, affected patients are often under- and mis-diagnosed due to low testing rates and poor understanding of the disease in non-endemic regions. In addition, HTLV-1 disease associations, including polymyositis, uveitis, thyroiditis, and bronchiectasis, are rarely considered unless patients have already been diagnosed with HTLV. This makes understanding HTLV and its relationships to other disease processes a significant challenge outside of regions of traditionally defined endemicity. Thus, we sought to better characterize the clinical features, comorbidities, and survival of HTLV-1- or HTLV-2-positive individuals identified in a non-endemic area, using patient information from our academic institution.

## Methods

### Study design and patient population

We conducted a retrospective, case–control study of patients with HTLV-1 or HTLV-2 newly diagnosed between January 1, 1998 and December 31, 2020 at a single institution. To obtain our patient group, we extracted data for patients with positive HTLV testing in our electronic health record and collected relevant patient level clinical information through direct chart abstraction. The diagnostic criteria for HTLV seropositivity in this study was defined by positive screening enzyme-linked immunoassay (EIA) or particle agglutination (PA) assays, with subsequent confirmation western blot or polymerase chain reaction (PCR) assays to discriminate between HTLV-1 and HTLV-2 seropositivity (Abrams et al., 2011). Using nearest neighbor propensity score matching on age, sex, and ethnicity, two HTLV seronegative patients were selected for each HTLV seropositive case. Patients were excluded from the study if they were less than 18 years of age or if no other clinical data was available other than their HTLV status. The protocol was approved by the institutional review board of Barnes Jewish Hospital (ID #202008022).

### Power and sample size determination

The sample size was calculated based on the prevalence of symptoms of HAM estimated at 3.7% of HTLV-1 positive individuals, with variance of 5%, based on a prior longitudinal study of HTLV-1/2-seropositive blood donors (Orland et al., 2003). In our medical center-based study, we expected to identify twice as many patients with HAM compared to a healthy, blood bank donor population. With 5% type I error and 80% power, we estimated that we would need 230 HTLV-1 positive patients to compare with historical control.

### Classification of HTLV comorbidities and co-infections

We collected relevant information on demographic, laboratory, and clinical variables from the electronic medical record database. Route of infection information including history of sexually transmitted infections, intravenous drug use, breast feeding in setting of known maternal infection, blood transfusions, and transplantation (including both solid organ and hematologic) were obtained. Reasons for HTLV testing were grouped into eight mutually exclusive categories: neurologic deficits, hematologic disorders, transplant evaluation, dermatological, gastrointestinal, lymphadenopathy, infection, and other/unknown. Co-infections with HIV, hepatitis B, or hepatitis C were evaluated from serological tests. HBV infection was defined by hepatitis B surface antigen (HBsAg), hepatitis B surface antibody (anti-HBs), and total hepatitis B core antibody (anti-HBc total) serologies. Opportunistic infections were defined by positive testing for CMV, EBV, Cryptococcus, Toxoplasmosis, bacterial blood stream, or *Clostridium difficile* infections.

We reviewed data on potential clinical manifestations of HTLV infection. Neurologic symptoms were defined by symptoms of lower extremity weakness and/or spastic paraparesis. Dermatologic symptoms were defined by eczematous, papular, or erythrodermic rash and/or confirmatory biopsy results. Ocular disturbances/uveitis included sudden onset of floaters, visual blurring, or discomfort. Pulmonary symptoms of pneumonitis were defined by computed tomography (CT) findings of parenchymal abnormalities, including centrilobular nodules, ground-glass opacities, and bronchiectasis (Dias et al., 2018). Comorbidities of rheumatoid arthritis, hypothyroidism, idiopathic thrombocytopenic purpura (ITP), malignancies, and mood disorders were also recorded.

### Statistical analysis

Standard descriptive statistics were used to describe the baseline characteristics of the population. Statistical comparisons between groups were performed with a chi-square test for categorical values and a *t*-test for numerical values. Univariate odds ratios (ORs) with 95% confidence intervals (CIs) were calculated with small sample adjustment using “epitools” in Rstudio. Overall survival (OS) was defined as elapsed time from diagnosis until death from any cause. Event-free patients were censored at date of last clinical follow-up. Survival estimates were calculated by Kaplan–Meier method and compared using the log-rank test. Both univariate and multivariate Cox regression models were fitted to assess clinical variables and OS. A two-sided *p* value <0.05 was considered statistically significant.

## Results

### Patient characteristics

We identified 160 patients with positive HTLV screening tests. Of these positive screening tests, 16 patients had no follow-up confirmatory western blot, 89 patients had negative western blots, and 16 patients had indeterminant western blots. There were 39 HTLV-seropositive patients confirmed with positive western blot, of whom 24 were HTLV-1 positive and 15 were HTLV-2 positive. One HTLV-1 positive patient was excluded who did not have any other clinical data available other than their HTLV status. The baseline patient characteristics are summarized in Table 1.

**Table 1:** Baseline patient demographics and characteristics.

	Total (*n* = 38)	HTLV-1 (*n* = 23)	HTLV-2 (*n* = 15)	value of *p*
Sex				
Males	21 (55%)	13 (57%)	8 (53%)	*p* = 0.85
Females	17 (45%)	10 (43%)	7 (47%)	
Age – yr				
Median ± SD	55 ± 11.0	55 ± 13.0	52 ± 6.7	*p* = 0.28
Range	18–81	18–81	39–66	
Ethnicity				
Caucasian	9 (24%)	4 (18%)	5 (33%)	*p* = 0.31
Black	25 (66%)	15 (65%)	10 (67%)	
Hispanic	1 (2%)	1 (4%)	0 (0%)	
Other	3 (8%)	3 (13%)	0 (0%)	
Risk factors				
IV drug use	9 (24%)	4 (17%)	5 (33%)	*p* = 0.25
STI	6 (16%)	4 (17%)	2 (13%)	*p* = 0.73
Transplant	6 (16%)	5 (22%)	1 (7%)	*p* = 0.60
Hx blood transfusion	2 (5%)	2 (9%)	0 (0%)	NA
Breastfeeding	1 (2%)	1 (4%)	0 (0%)	NA
None identified	14 (37%)	7 (31%)	7 (47%)	*p* = 0.49
HIV				
Positive	0 (0%)	0 (0%)	0 (0%)	NA
Negative	34 (89%)	21 (91%)	13 (87%)	
Unknown	4 (11%)	2 (9%)	2 (13%)	
Hepatitis C Virus				
Positive	11 (29%)	3 (13%)	8 (54%)	*p* = 0.0074
Negative	21 (55%)	16 (70%)	5 (33%)	
Unknown	6 (16%)	4 (17%)	2 (13%)	

Statistical comparisons between HTLV-1 and HTLV-2 cases were performed with a chi-square test or Fischer’s Exact Test for categorical and a *t*-test for numerical values.

The distribution of matching factors—age, sex, and ethnicity—was similar between HTLV seropositive patients and seronegative controls. Compared to control population, the HTLV seropositive population had higher rates of intravenous drug use (IVDU) (Supplementary Table S1), which is consistent with previous studies and transmission of HTLV through sharing of blood-contaminated needles (Murphy et al., 1999). In addition, HTLV-2 patients had higher rates of IVDU compared to HTLV-1 patients (Table 1). Other risk factors, including sexually transmitted infections, history of blood transfusions, and breastfeeding were not identified by our study.

### Reasons for HTLV testing

The reasons for HTLV testing are identified in Table 2.

**Table 2:** Summary of reasons for HTLV testing observed for HTLV-seropositive patients and controls.

	HTLV-seropositive patients (*n* = 38)	Controls (*n* = 76)
Transplant evaluation	9 (23.7%)	41 (53.9%)
Neurologic deficit	12 (31.5%)	19 (25%)
Hematologic disorder	5 (13.2%)	7 (9.2%)
Dermatologic disorder	1 (2.6%)	1 (1.3%)
Gastrointestinal	1 (2.6%)	0 (0%)
LAD	1 (2.6%)	0 (0%)
Infection	0 (0%)	1 (1.3%)
Other/Unknown	9 (23.7%)	7 (9.2%)

HTLV-seropositive patients predominantly underwent HTLV testing because of neurologic deficits (31.5%)—including spastic paraparesis, weakness, and gait disturbances. The second most common reason for HTLV testing was for transplant screening tests. HTLV screening was included in the routine serologic panel obtained prior to transplant and often was diagnosed incidentally in asymptomatic patients. Notably, the majority (53.9%) of patients in our control group received HTLV testing for transplant evaluation testing, compared to 23.7% of HTLV-seropositive patients.

### Comparisons of malignancies and transplant

Due to the increased representation of transplant evaluation patients in the control group, we sought to further evaluate the prevalence of malignancies, solid/hematologic transplants, and potential biases in survival in our study population.

We found that there was no difference in the frequency of solid or hematologic malignancies between HTLV seropositive patients and the control population (Table 3). Eight patients with HTLV-1 had diagnoses of ATL. When excluding these patients with ATL (which requires prior HTLV-1 infection), we found that our control group was enriched in patients with hematologic malignancies, with 37 of 76 in the control population with hematologic malignancies compared to 7 of 38 in the patient population (*p* = 0.0021). In particular, around half of the hematologic malignancies in the control population (17/37, or 46%) were multiple myeloma.

**Table 3:** Frequency of solid and hematologic malignancies for HTLV-seropositive patients and controls.

	HTLV-seropositive (*n* = 38)	Controls (*n* = 76)	value of *p*
Cancer	21/38 (55%)	42/76 (55%)	*p* = 1.0
Solid	7 (19%)	10 (13%)	*p* = 0.46
Lung	1 (3%)	1 (1%)	
Liver	2 (5%)	0 (0%)	
Colon	0 (0%)	2 (3%)	
Breast	1 (3%)	4 (5%)	
Gyn	2 (5%)	0 (0%)	
Skin	1 (3%)	1 (1%)	
Prostate	0 (0%)	2 (3%)	
Heme	15 (39%)	37 (49%)	*p* = 0.35
ATL	8 (21%)	0 (0)	
AML	2 (5%)	7 (9%)	
MM	0 (0%)	17 (22%)	
Other	5 (13%)	13 (17%)	
Transplant			
Solid	3 (8%)	8 (11%)	*p* = 0.96
Heme	5 (13%)	28 (37%)	*p* = 0.0085
Allogeneic	3 (%)	8 (%)	*p* = 0.65
Autologous	3 (%)	20 (71%)	*p* = 0.0080

Statistical comparisons between HTLV-seropositive cases and control were performed with a *t*-test for numerical values.

Moreover, our study control population had increased rates of autologous stem cell transplant (auto-SCT) compared to our HTLV patient population, likely reflecting the increased representation of multiple myeloma patients. There was an insignificant difference in allogeneic stem cell transplants (allo-SCT) and solid organ transplants. In our HTLV-seropositive patient population, there was one patient who underwent both auto- and allo-SCT for relapsed and refractory DLBCL. In our control population, one patient underwent both solid organ deceased donor kidney transplant and auto-SCT for multiple myeloma.

**Figure 1:**
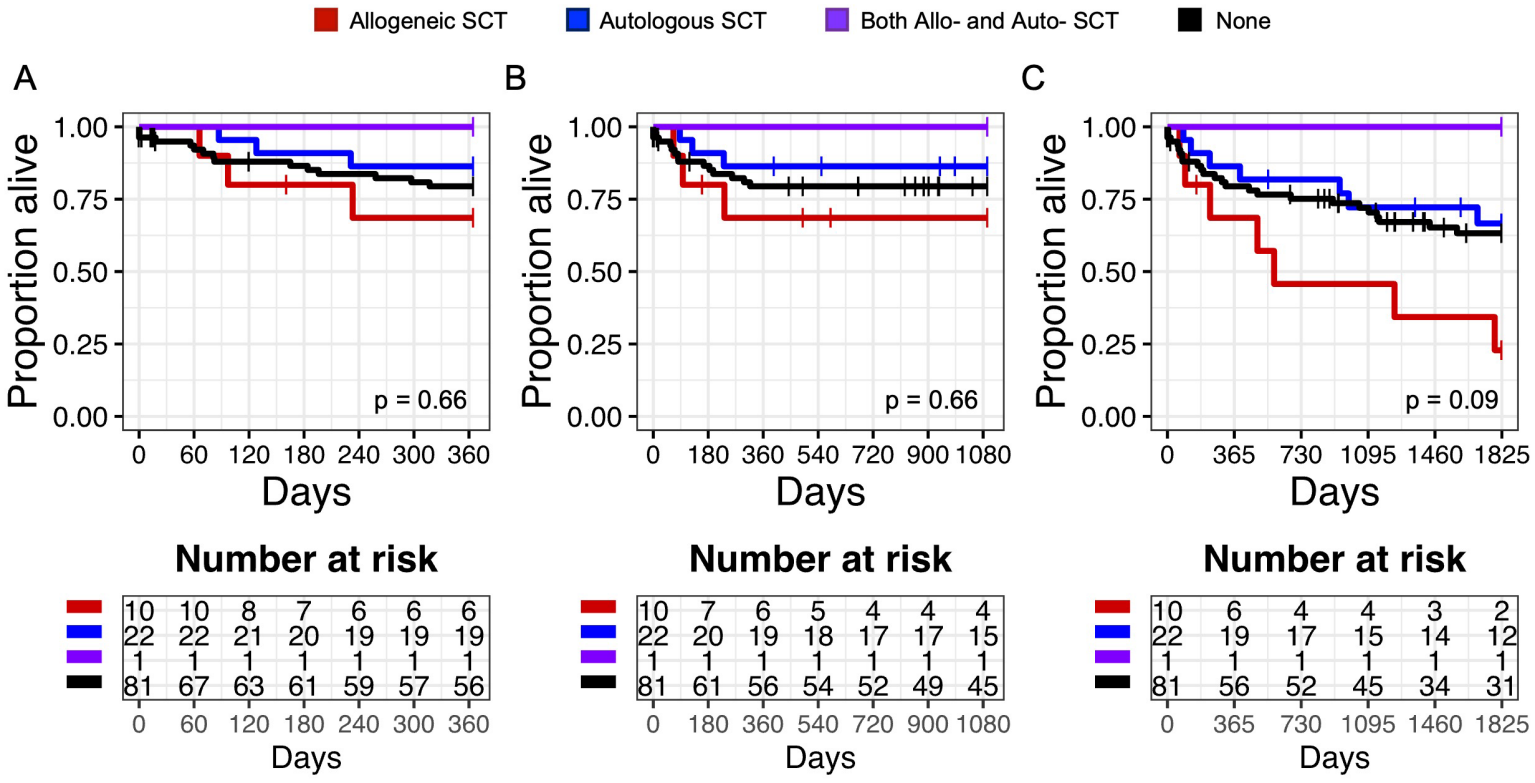
Overall Survival (OS) in patients with a history of hematologic transplant. Kaplan Meier graphs of survival probability for subjects (both HTLV-seropositive and control) who received allogeneic stem cell transplantation (SCT) (red line), autologous SCT (blue line), both allo- and auto-SCT (purple line), or none (black line) at 1 year **(A)**, 3 years **(B)**, and 5 years **(C)**. Vertical marks represent censored events.

To ensure that this increase in autologous transplants would not bias our outcomes analysis, we evaluated the impact of transplant on survival. In Kaplan Meier analysis, there was no difference in overall survival at 1 year (*p* = 0.66), 3 years (p = 0.66), or 5 years (*p* = 0.09) in patients who had history of allo-SCT, auto-SCT, both allo-SCT and auto-SCT, or no transplants (Figure 1).

There was similarly no difference in survival in patients with solid organ transplants at 1 year (*p* = 0.87), 3 years (*p* = 0.87), or 5 years (*p* = 0.53) after HTLV diagnosis (Supplementary Figure S1).

### Clinical manifestations and comorbidities associated with HTLV seropositivity

**Figure 2:**
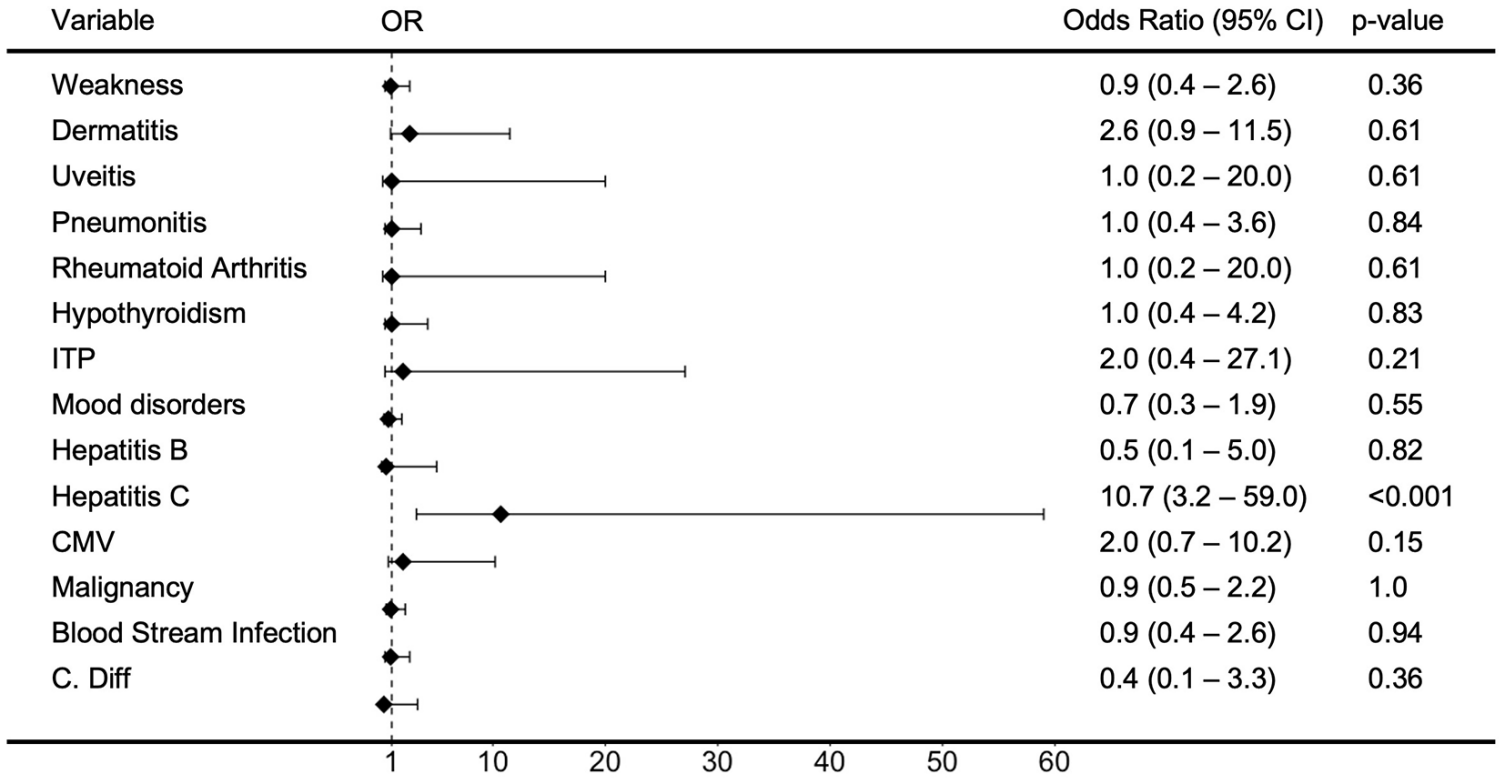
Odds Ratio (OR) of clinical variables in patients with HTLV infection. Forest Plot and analysis showing the Odds Ratio of various clinical covariates in patients with HTLV infection. The black diamonds and lines indicate the odds ratios (ORs) and their confidence intervals (CIs) for each variable. Odds ratios and confidence intervals were calculated by small sample adjustment method.

When evaluating for clinical manifestations of HTLV in chart review, neurologic symptoms of lower extremity weakness or spastic paraparesis were observed in 24% (*n* = 9), with only three cases directly attributed to HTLV-1 associated myelopathy (HAM) (*n* = 1) or HTLV-2 spastic myelopathy (*n* = 2) (Supplementary Table S2). Other clinical manifestations included dermatitis in 16% (*n* = 6), ocular disturbances/uveitis in 3% (*n* = 1), and pneumonitis in 13% (*n* = 5) of patients. Comorbidities evaluated included rheumatoid arthritis (3%, *n* = 1), hypothyroidism (11%, *n* = 4), ITP (5%, *n* = 2), malignancies (55%, *n* = 21), and mood disorders (24%, *n* = 9). Odds ratios for these clinical manifestations and comorbidities were not statistically significant for HTLV-seropositivity when compared to controls (Figure 2). Further odds ratio analysis separated by HTLV-1 and HTLV-2 infection found an association of dermatitis with HTLV-1 infection (OR 3.8, 95% CI 1.3–16.9, *p* = 0.027) and weakness with HTLV-2 infection (OR 2.4, 95% CI 0.9–8.6, *p* = 0.020) (Supplementary Figures S2, S3).

Notably, there were no cases of co-infection with HIV in our patient population.

When evaluating for hepatitis B virus infection, we found that in our patient population, 5% (*n* = 2) had prior HBV infection, 74% (*n* = 28) were negative, and 21% (*n* = 8) had not undergone complete hepatitis B serologic testing. HCV antibodies were observed in 29% (*n* = 11) of the patient population. Hepatitis C seropositivity was strongly associated with HTLV-seropositivity (OR 10.7, 95% CI 3.2–59.0, *p* < 0.001) (Figure 2) and HTLV-2 was associated with higher risk of Hepatitis C seropositivity than HTLV-1 (OR 17.8, 95% CI 5.7–89.7, *p* < 0.001 and OR 1.1, 95% CI 0.4–5.3, *p* = 0.71) (Supplementary Figures S2, S3). Other co-infections included CMV (*n* = 7), blood stream infections (*n* = 9), Clostridioides difficile (C. diff) (*n* = 1), and strongyloidiasis (*n* = 1), but these were not associated with higher risk with HTLV-seropositivity, HTLV-1 infection, or HTLV-2 infection.

### Outcomes

Median follow-up was 39.7 months and median OS was 67.2 months for HTLV-seropositive patients, compared to 49.6 months and 131.2 months, respectively, for HTLV-seronegative patients. HTLV-1 positive patients had lower median OS compared to HTLV-2 patients (47.7 months vs. 77.4 months). During follow-up, 21 of 38 (55.3%) HTLV-seropositive patients died, while 33 of 76 (43.4%) HTLV-seronegative patients died.

In univariate analysis, the hazard for 1 year all-cause mortality was increased among patients with HTLV-seropositivity (unadjusted HR 3.4, 95% CI 1.4–8.2, *p* = 0.006), HTLV-1 infection (unadjusted HR 2.4, 95% CI 1.0–6.0, *p* = 0.044), ATL (unadjusted HR 4.8, 95% CI 1.8–13.3, *p* = 0.08), AML (unadjusted HR 4.3, 95% CI 1.6–11.9, *p* = 0.01), and HCV infection (unadjusted HR 3.2, 95% CI 1.2–8.5, *p* = 0.03) (Table 4). When corrected, multivariate analysis showed that HTLV seropositivity was no longer associated with 1 year all-cause mortality; however association with AML (adjusted HR 10.0, 95% CI 2.8–35.2, *p* = 0.0003) and HCV infection (adjusted HR 7.0, 95% CI 1.5–32.0, *p* = 0.01) remained significant.

**Table 4:** Cox regression model of clinical factors associated with 1 year overall survival (OS).

Clinical factor	Univariate analysis		Multivariate model	
	HR (95% CI)	value of *p*	HR (95% CI)	value of *p*
HTLV seropositivity	3.4 (1.4–8.2)	0.0068	1.6 (0.5–5.4)	0.46
HTLV-1	2.4 (1.0–6.0)	0.044	NA	NA
HTLV-2	2.2 (0.8–5.9)	0.13	NA	NA
IV drug use	2.7 (1.0–7.4)	0.051	0.6 (0.2–2.4)	0.49
Hematologic malignancy				
ATL	4.8 (1.8–13.3)	0.0021	4.8 (0.9–26.1)	0.068
AML	4.3 (1.6–11.9)	0.0044	10.0 (2.8–35.2)	<0.001
MM	0.2 (0.03–1.7)	0.15	NA	NA
Other	0.9 (0.3–3.0)	0.86	NA	NA
Solid malignancy	0.9 (0.3–2.9)	0.82	NA	NA
Hepatitis C	3.2 (1.2–8.5)	0.017	7.0 (1.5–32.0)	0.012
Blood stream infection (BSI)	1.3 (0.5–3.5)	0.59	NA	NA

Data for the univariate analysis included relevant predictor variables, including patient characteristics, malignancies, and co-infections. Clinically and statistically significant variables from the univariate analysis were then included for analysis in the multivariate model. “NA” indicates values that were not included in the multivariate model.

**Figure 3:**
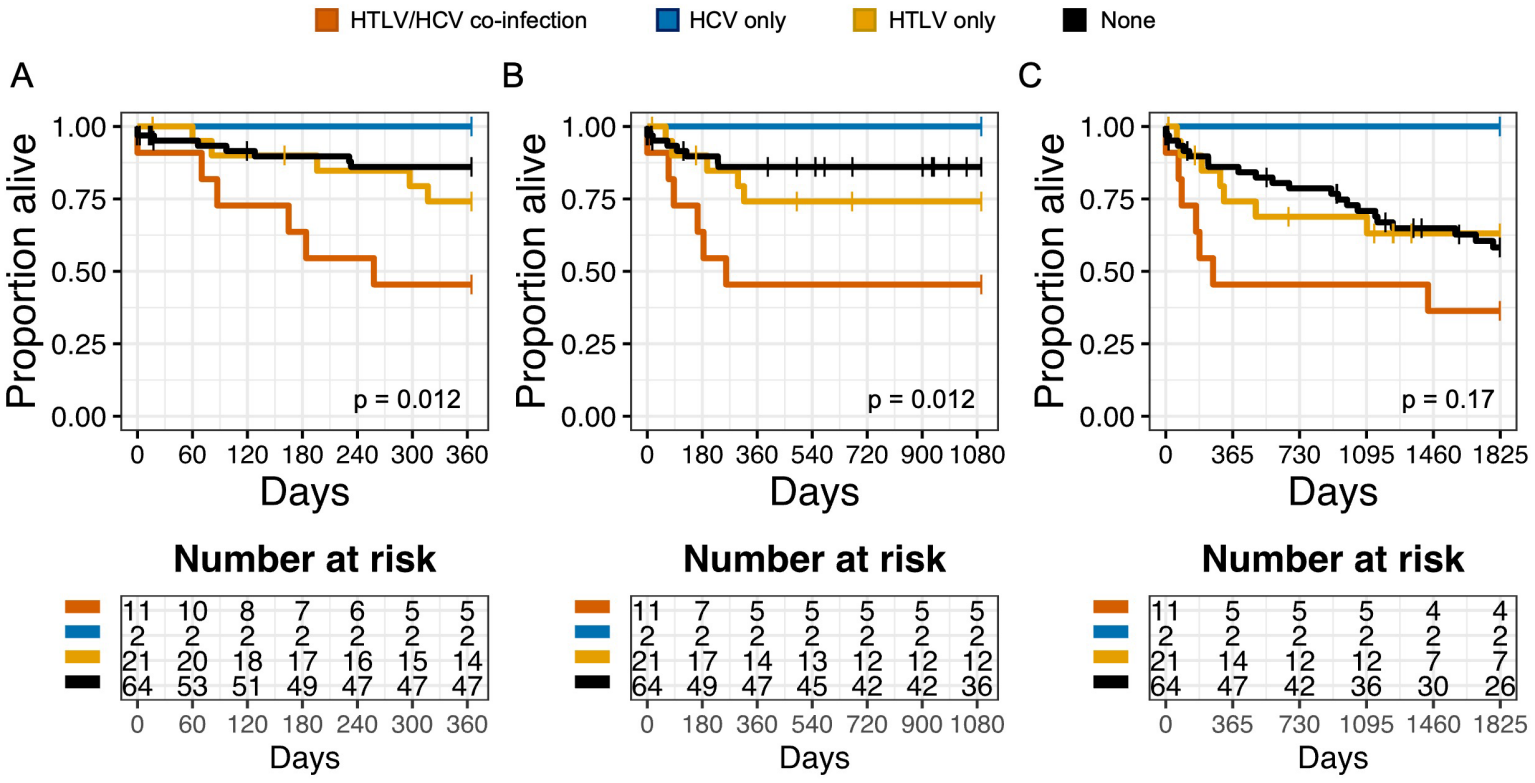
Co-infection with HTLV negatively impacts overall survival (OS) in patients with Hepatitis C virus (HCV) infection. Kaplan Meier graphs of survival probability for patients stratified by HTLV/Hepatitis C (HCV) co-infection (orange), HCV only (blue), HTLV only (yellow), or none (black) at 1 year **(A)**, 3  years **(B)**, and 5  years **(C)**. Vertical marks represent censored events.

In Kaplan Meier analysis, HCV and HTLV co-infection resulted in decreased 1 year and 3 year OS, compared to no infection, HCV infection alone, or HTLV infection alone (Figures 3A–C). This suggested that patients with HCV and HTLV co-infection had worse outcomes compared to infection with HCV or HTLV alone. When we performed survival analysis examining HTLV-1 and HTLV-2 infection separately, we found that while HCV and HTLV-2 co-infection was associated with worse 1 year and 3 year OS compared to no infection, HCV infection alone, or HTLV-2 infection alone, this was not true for HTLV-1 and HCV co-infection (Supplementary Figures S4A–F).

**Figure 4:**
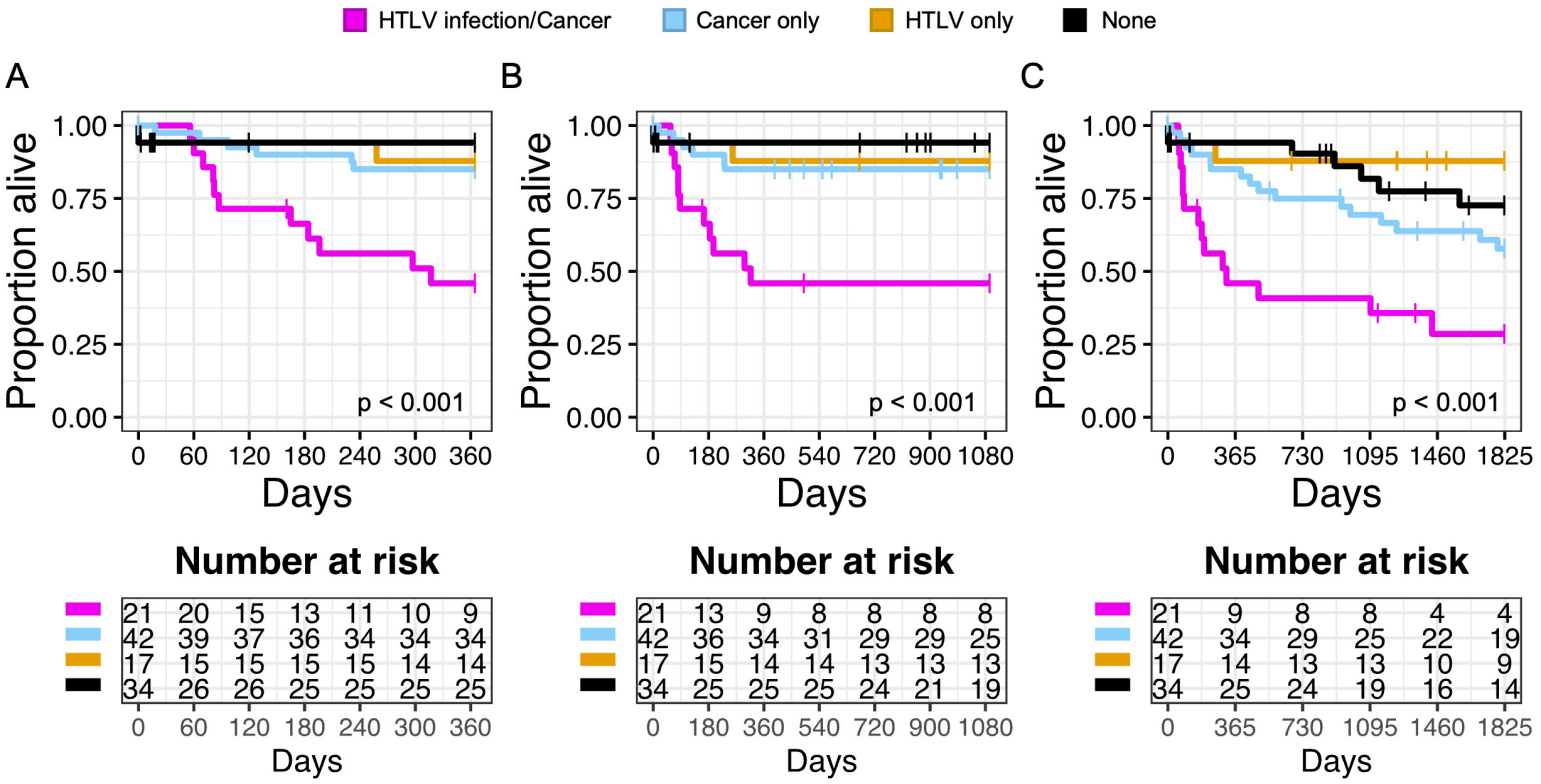
Infection with HTLV negatively impacts overall survival (OS) in patients with cancer. Kaplan Meier graphs of survival probability for patients stratified by HTLV/Cancer (magenta), cancer only (light blue), HTLV only (yellow), or none (black) at 1 year **(A)**, 3 years **(B)**, and 5 years **(C)**. Dashed lines represent censored events.

Similarly, patients with any cancer diagnosis and HTLV infection had worse 1 year, 3 year, and 5 year OS compared to patients with cancer or HTLV alone (Figures 4A–C). This was also true in subgroup analysis when we examined patients with any cancer diagnosis and HTLV-1 or HTLV-2 infection (Supplementary Figures S5A–F). There was no significance in 1 year OS with other risk factors, including CMV/HTLV co-infection or bacterial blood stream/HTLV co-infection (Supplementary Figures S6A,B).

## Discussion

Since the discovery of HTLV-1 as the first oncogenic virus in 1980, our understanding of HTLV infection remains largely based on limited epidemiologic and clinical data from known endemic areas. With globalization, more and more PLHTLV have migrated from endemic to non-endemic areas, and recent studies have shown a rise of HTLV infection in the United States. As the majority of infected individuals remain asymptomatic, diagnosing and managing an infrequently encountered disease remains a significant challenge for many physicians in non-endemic areas. We sought to better characterize HTLV from data from our institution and identified 38 cases of HTLV-seropositive patients.

Our study supports previous findings of increased prevalence of intravenous drug use and hepatitis C virus (HCV) co-infection in the HTLV seropositive population (Murphy et al., 1999). Epidemiologic data from Japan and Brazil (in regions endemic for HTLV) have reported HCV co-infection rates in HTLV-1/2 seropositive patients as high as 35.9%, and have suggested transmission routes through transfusions of infected blood products and intravenous drug use (Nakashima et al., 1994; Pinto et al., 2012; Pereira et al., 2020). A recent meta-analysis of three separate studies of HTLV/HCV co-infection from China, Brazil, and Sweden found a pooled OR of 20.1 (Rosadas and Taylor, 2022). In our study, individuals with HTLV were 10.7 times more likely to be co-infected with HCV, and HTLV-2 seropositive individuals were more likely to have HCV co-infection than HTLV-1 seropositive individuals. Our study results are consistent with previous findings from Murphy et al.’s screening of blood bank donors, which reported an increased risk of HCV seropositivity with HTLV-1 (OR 5.3, 95% CI 2.6–11.1) and HTLV-2 (OR 25.0, 95% CI 18.7–32.4) (Murphy et al., 1999).

Notably, in our evaluation of clinical covariates, we found that HTLV seropositivity was associated with increased all-cause mortality in univariate analysis, but when corrected, this was no longer true. Rather, all-cause mortality appears to have been driven by other diseases, including HCV infection and AML. A seminal meta-analysis by Schierhout et al. examining epidemiologic associations between HTLV and adverse health outcomes reported that HTLV-1 infection was associated with increased risk of premature death, independent of ATL or HAM (RR 1.57, 95% CI 1.37–1.80). The authors theorize that given the dearth of evidence for fatal comorbidities with HTLV-1 that would contribute to this increased mortality, HTLV-1 infection may cause systemic effects that have not yet been sufficiently studied or understood (Schierhout et al., 2020).

Interestingly, our study found that HTLV/HCV co-infection resulted in decreased OS compared to HCV or HTLV infection alone. Several studies from Japan found similar results and showed worsening HCV viremia, more rapid progression of liver disease to cirrhosis and hepatocellular carcinoma, and decreased response to treatment in patients with HTLV/HCV co-infection (Nakashima et al., 1994; Kishihara et al., 2001). Another recent large cohort study from Brazil similarly found HIV and HCV coinfection to be independent predictors of mortality in PLHTLV (HR 15.1, 95% CI 5.5–41.3) (Marcusso et al., 2019). This has been hypothesized to be due to the impaired cellular immune responses in HTLV-1-infected T-cells mediated by HTLV-1 bZIP factor (HBZ) induced expression of co-inhibitory molecules (such as PD-1, CTLA-4, and TIGIT) and production of inhibitory cytokines like interleukin-10 (IL-10), leading to ineffective resolutions of infections (Kozako et al., 2009; Sawada et al., 2017; Higuchi et al., 2020; Tan et al., 2022). Interestingly, the immunomodulating effects of HTLV-2 infection are less well understood, with recent studies of HIV/HTLV-2 co-infection suggesting that HTLV-2 may exert a protective effect on survival and slowing rate of disease progression to AIDS, although these results are mixed (Giacomo et al., 1995; Hershow et al., 1996; Castro-Sansores et al., 2006; Bassani et al., 2007).

Similarly, our study determined that patients with either HTLV-1 or HTLV-2 infection and a cancer diagnosis had worse OS than patients with cancer alone. Studies of PLHTLV have implicated HTLV-1 in various cancers and its promotion of cancer progression (Arisawa et al., 2003; Stienlauf et al., 2013; Dias et al., 2018; Du et al., 2019). Indeed, our findings are consistent with recent retrospective cohort data from Valcarcel et al. showing that PLHTLV with concomitant non-ATL cancer diagnoses, excluding diffuse large B-cell lymphoma (DLBCL), had worse survival outcomes than those reported in the general Latin American population (Valcarcel et al., 2022, 2023). While the exact mechanism contributing to worse survival outcomes is still under debate, data have suggested that CD4+ T cell dysfunction mediated by HTLV infection may contribute to defects in anti-tumor responses (Höllsberg et al., 1992; Mueller et al., 1996; Sibon et al., 2006). Some investigators have also suggested that the chronic inflammatory microenvironment induced by HTLV-infection can contribute to tumorigenesis. *In vivo* laboratory studies of HTLV-1 have shown that even asymptomatic PLHTLV have higher serum levels of inflammatory cytokines (including, IL-2, IL-6, TNF-alpha, IFN-gamma) compared to healthy controls (Montanheiro et al., 2009; Domingos et al., 2017). HTLV-1 related T-cell activation is believed to be induced by the viral protein Tax, which leads to p53 tumor pathway inactivation and subsequent downstream dysregulation of apoptosis, DNA stability, and cell proliferation (Tornesello et al., 2018). Thus, Tax activation influences many downstream pathways separate from its involvement in ATL, including other cancers like small cell lung cancer, AML, pancreatic cancer, and colorectal cancer.

It must be noted that our study did not identify any HIV/HTLV co-infected patients. This was unexpected given the high frequency of HIV/HTLV co-infection noted in the literature (Beilke, 2012; de Mendoza et al., 2019). We hypothesize that this may be due to the low testing rates for HTLV in our hospital environment—as HTLV remains a rare and neglected disease in our region, very few providers would think to test for asymptomatic HTLV in HIV-infected patients. Indeed, HTLV was predominantly diagnosed incidentally in our patient population as part of a standardized transplant evaluation. Given that even asymptomatic HTLV infection may worsen clinical disease courses, we believe this warrants routine screening for HTLV in high-risk populations in non-endemic areas.

We also acknowledge the limitation of grouping together HTLV-1 and HTLV-2 seropositive patient populations in our study. While we performed subgroup analysis of HTLV-1 and HTLV-2 infections in our study, we acknowledge that these results are difficult to interpret given the small patient population in each group. Due to incomplete laboratory follow-up and infrequent testing for HTLV at our institution, our study was limited by our small patient sample size as well as the biased patient control population.

Our study failed to identify clinical characteristics of HTLV infection (including uveitis, pneumonitis, thyroiditis, or mood disturbances) which have been described in prior larger studies. This may largely be attributed to the fact that our study was underpowered. For example, to detect HTLV-associated myelopathy (HAM), our power calculation indicated that we would need to identify 230 HTLV-1 patients, however, our study was only able to obtain records from 24 HTLV-1 patients, three of whom had a formal diagnosis of HAM. Interestingly, nine of 39 HTLV-seropositive patients had neurological symptoms of lower extremity weakness and/or spastic paraparesis—it is possible that many of these cases may have been misdiagnosed due to the rarity of HAM and its clinical overlap with other neurological conditions, including multiple sclerosis, nutritional deficiencies, primary lateral sclerosis, and other inflammatory disorders (De et al., 2006; Oh and Jacobson, 2008).

Future studies with a larger patient cohort are critical to further evaluate co-morbidities and associated disease characteristics of HTLV-1 and HTLV-2 seropositive patients.

## Data availability statement

The original contributions presented in the study are included in the article/Supplementary material, further inquiries can be directed to the corresponding author.

## Ethics statement

The studies involving human participants were reviewed and approved by Washington University Institutional Review Board. Written informed consent for participation was not required for this study in accordance with the national legislation and the institutional requirements.

## Author contributions

MG, TT, and LR conceived and designed the study, and wrote the manuscript. SP, TT, and LR designed the protocol and submitted the IRB. MG collected, analyzed, and assembled the data. FG verified the analytical methods. All authors contributed to the article and approved the submitted version.

## Funding

This project was supported by the Washington University Institute of Clinical and Translational Sciences which is, in part, supported by the NIH/National Center for Advancing Translational Sciences (NCATS), CTSA grant #UL1 TR002345. Funding was also provided through the Washington University Clinical- Scientist Training and Research (C-STAR)/Mentors in Medicine (MiM) Program. This project was also funded in part by the Alvin J. Siteman Cancer Center and Barnes-Jewish Hospital. The Siteman Cancer Center is supported in part by an NCI Cancer Center Support Grant #P30 CA091842. Additional support was provided by the Barnard Cancer Institute.

## Conflict of interest

The authors declare that the research was conducted in the absence of any commercial or financial relationships that could be construed as a potential conflict of interest.

The reviewer JCR declared a past collaboration with the author LR to the handling editor.

## Publisher’s note

All claims expressed in this article are solely those of the authors and do not necessarily represent those of their affiliated organizations, or those of the publisher, the editors and the reviewers. Any product that may be evaluated in this article, or claim that may be made by its manufacturer, is not guaranteed or endorsed by the publisher.
