# Supplementary material for: Clinical characteristics and outcomes of infection with human T-lymphotropic virus in a non-endemic area: a single institution study
Source: Front Microbiol. 2023 Jun 22;14:1187697. doi: 10.3389/fmicb.2023.1187697 (PMC10324566; doi:10.3389/fmicb.2023.1187697)
Supplement: Supplementary file 1 [file Data_Sheet_1.PDF]

Supplemental Table and Figures for

Clinical Characteristics and Outcomes of Infection with Human T-cell Lymphotropic Virus (HTLV) in a Non-endemic Area: A Single Institution Study

Margery Gang, Feng Gao, Sneha Poondru, Theodore Thomas, Lee Ratner

Supplemental Table 1: Baseline patient demographics and characteristics of HTLV-seropositive patients and control

	HTLV-seropositive (N = 38)	Control (N = 76)	P-value
**Sex**			p = 0.69
Male	17 (45%)	37 (49%)	
Female	21 (55%)	39 (51%)	
**Age – yr**			p = 0.72
Median ± SD	55 ± 11.0	55 ± 10.7	
Range	18 - 81	18 - 78	
Race			p = 0.99
Caucasian	9 (24%)	19 (24%)	
Black	22 (58%)	51 (67%)	
Hispanic	1 (3%)	2 (3%)	
Other	3 (8%)	5 (7%)	
Risk factors			
IVDU	9 (24%)	3 (4%)	p = 0.001
STI	6 (16%)	19 (25%)	p = 0.26
Transplant	6 (16%)	32 (42%)	p = 0.28
Hx blood tx	2 (5%)	16 (21%)	p = 0.030
Breastfeeding	1 (3%)	NA	NA
HIV			
Positive	0 (0%)	1 (1%)	NA
Negative	34 (89%)	70 (92%)	
Unknown	4 (11%)	5 (7%)	
Hepatitis C Virus			
Positive	11 (29%)	2 (3%)	$p < 0.001$
Negative	21 (55%)	64 (84%)	
Unknown	6 (16%)	10 (13%)	
Cancer type			
Solid	7 (19%)	10 (13%)	p = 0.46
Lung	1 (3%)	1 (1%)	
Liver	2 (5%)	0 (0%)	
Colon	0 (0%)	2 (3%)	
Breast	1 (3%)	4 (5%)	
Gyn	2 (5%)	0 (0%)	
Skin	1 (3%)	1 (1%)	
Prostate	0 (0%)	2 (3%)	
Heme	15 (39)	37 (49%)	p = 0.35
ATLL	8 (21)	0 (0)	
AML	2 (5)	7 (9%)	
MM	0 (0)	17 (22%)	
Other	5(13)	13 (17%)	

Supplemental Table 2: Neurologic presentations of lower extremity weakness and/or spastic paraparesis in HTLV-seropositive patients

Patient #	Age	Sex	Ethnicity	HTLV serotype	History and/or clinical findings	Chart diagnosis
1	51	F	Black	1	Non-spastic lower extremity paralysis and bladder/bowel incontinence	Tumor involvement of CNS (supported by PET imaging)
2	48	F	Black	2	Spastic lower extremity paralysis	HAM
3	78	F	Black	1	10 year history of progressive spastic paraparesis	HAM
4	62	F	Black	1	Spastic lower extremity paralysis and neurogenic bladder. Previously followed with neurologist, however, lost to follow-up.	"Hereditary spastic paraparesis of unknown etiology"
5	66	F	Black	2	>20 year history of gait and balance issues. Physical exam with asymmetric hyperreflexia and increased tone in lower extremities	Myasthenia Gravis
6	52	F	Black	2	Subacute presentation of right lower extremity pain and paresis. Physical exam notable for "flaccid and hypo-reflexive" right lower extremity.	West Nile Virus (WNV)-associated flaccid paralysis (confirmed with WNV IgG and IgM testing)
7	49	M	Caucasian	2	Spastic lower extremity paralysis and bladder incontinence	HTLV-2 spastic myelopathy and neurogenic bladder
8	55	F	Black	2	Bilateral lower extremity weakness, saddle anesthesia, and urinary retention	Multiple sclerosis
9	39	F	Caucasian	2	Several month history of paresthesias in bilateral lower extremities that progressed acutely to lower extremity paralysis. Weakness eventually improved and regained ability to walk.	Leber's hereditary optic neuropathy plus syndrome (confirmed by genetic testing)

HAM = HTLV-1-associated myelopathy.

Supplemental Figure Legends

Supplemental Figure 1: OS stratified by solid organ transplant history. Kaplan Meier graphs of survival probability for patients with (blue dashed line) or without (black solid line) a history of solid-organ transplant at (A) one-, (B) three-, or (C) five- years. Vertical marks represent censored events.

Supplemental Figure 2: OR of clinical variables in patients with HTLV-1 infection. Forest Plot and analysis showing the OR of clinical covariates in patients with HTLV-1 infection. The black diamonds and lines indicate the odds ratios (ORs) and their confidence intervals (CIs) for each variable. Odds ratios and confidence intervals were calculated by small sample adjustment method.

Supplemental Figure 3: OR of clinical variables in patients with HTLV-2 infection. Forest Plot and analysis showing the OR of clinical covariates in patients with HTLV-2 infection. The black diamonds and lines indicate the odds ratios (ORs) and their confidence intervals (CIs) for each variable. Odds ratios and confidence intervals were calculated by small sample adjustment method.

Supplemental Figure 4: OS subgroup analysis of patients with HCV infection co-infected with HTLV-1 and HTLV-2. Kapan Meier graphs of survival probability for patients co-infected with (A – C) HTLV-1 and (D – F) HTLV-2 in patients with HCV. Survival was stratified by HTLV-1/2/HCV co-infection (line-dot), HTLV-1/2 only (long dash), HCV only (dotted line), or none (solid) at (A, D) 1-year, (B, E) 3-years, and (C, F) 5-years. Vertical marks represent censored events.

Supplemental Figure 5: OS subgroup analysis of patients with cancer and infection with HTLV-1 and HTLV-2. Kapan Meier graphs of survival probability for patients infected with (A – C) HTLV-1 and (D – F) HTLV-2 in patients with any cancer diagnosis. Survival was stratified by HTLV-1/2/cancer (line-dot), HTLV-1/2 only (long dash), cancer only (dotted line), or none (solid) at (A, D) 1-year, (B, E) 3-years, and (C, F) 5-years. Vertical marks represent censored events.

Supplemental Figure 6: Co-infection with other risk factors, including CMV and bacterial blood stream infections (BSIs), do not increase 1-year OS. Kaplan Meier graphs of 1-year survival probability for patients with HTLV and (A) CMV and (B) BSI infections. For (A), survival was stratified by HTLV/CMV co-infection (line-dot), CMV only (long dash), HTLV (dotted line), or none (solid). For (B), survival was stratified by HTLV/BSI co-infection (line-dot), BSI only (long dash), HTLV (dotted line), or none (solid). Vertical marks represent censored events.

SFig 1

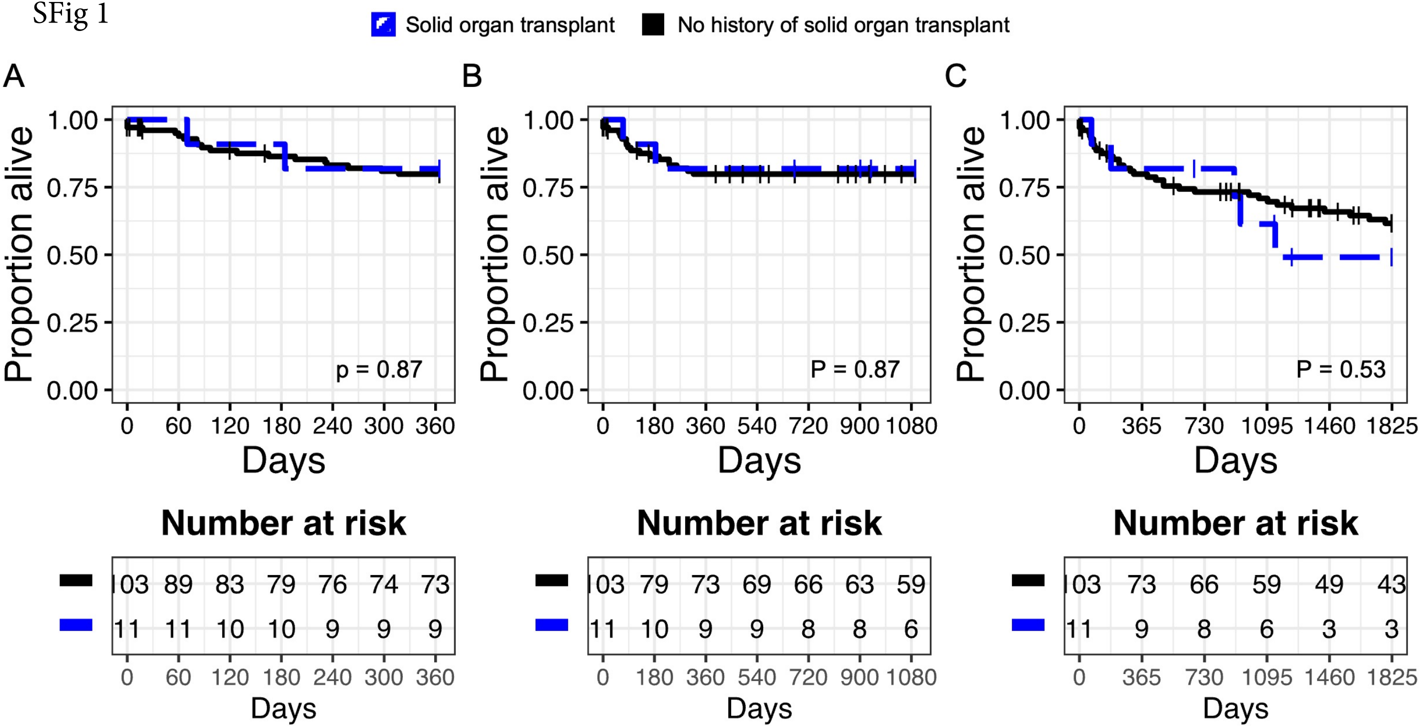
Solid organ transplant
No history of solid organ transplant
A
B
C
Proportion alive
1.00
0.75
0.50
0.25
0.00
p = 0.87
P = 0.87
P = 0.53
0 60 120 180 240 300 360
0 180 360 540 720 900 1080
0 365 730 1095 1460 1825
Days
Number at risk
103 89 83 79 76 74 73
11 11 10 10 9 9 9
103 79 73 69 66 63 59
11 10 9 9 8 8 6
103 73 66 59 49 43
11 9 8 6 3 3

Variable	Odds Ratio (95% CI)	p-value
Weakness	0.4 (0.1 – 1.6)	0.42
Dermatitis	3.8 (1.3 – 16.9)	0.027
Uveitis	0.0 (0.4 – 16.4)	0.47
Pneumonitis	1.5 (0.5 – 6.0)	0.48
Rheumatoid Arthritis	0.0 (0.4 – 16.4)	1.0
Hypothyroidism	1.3 (0.4 – 6.3)	0.69
ITP	4.1 (0.9 – 56.1)	0.10
Mood disorders	0.5 (0.2 – 1.7)	0.30
Hepatitis B	0.0 (0.0 – 3.8)	0.29
Hepatitis C	1.1 (0.4 – 5.3)	0.71
CMV	3.0 (1.0 – 15.6)	0.066
Malignancy	1.5 (0.6 – 4.2)	0.35
Blood Stream Infection	1.0 (0.4 – 3.4)	1.0
C. Diff	0.8 (0.2 – 10.3)	1.0

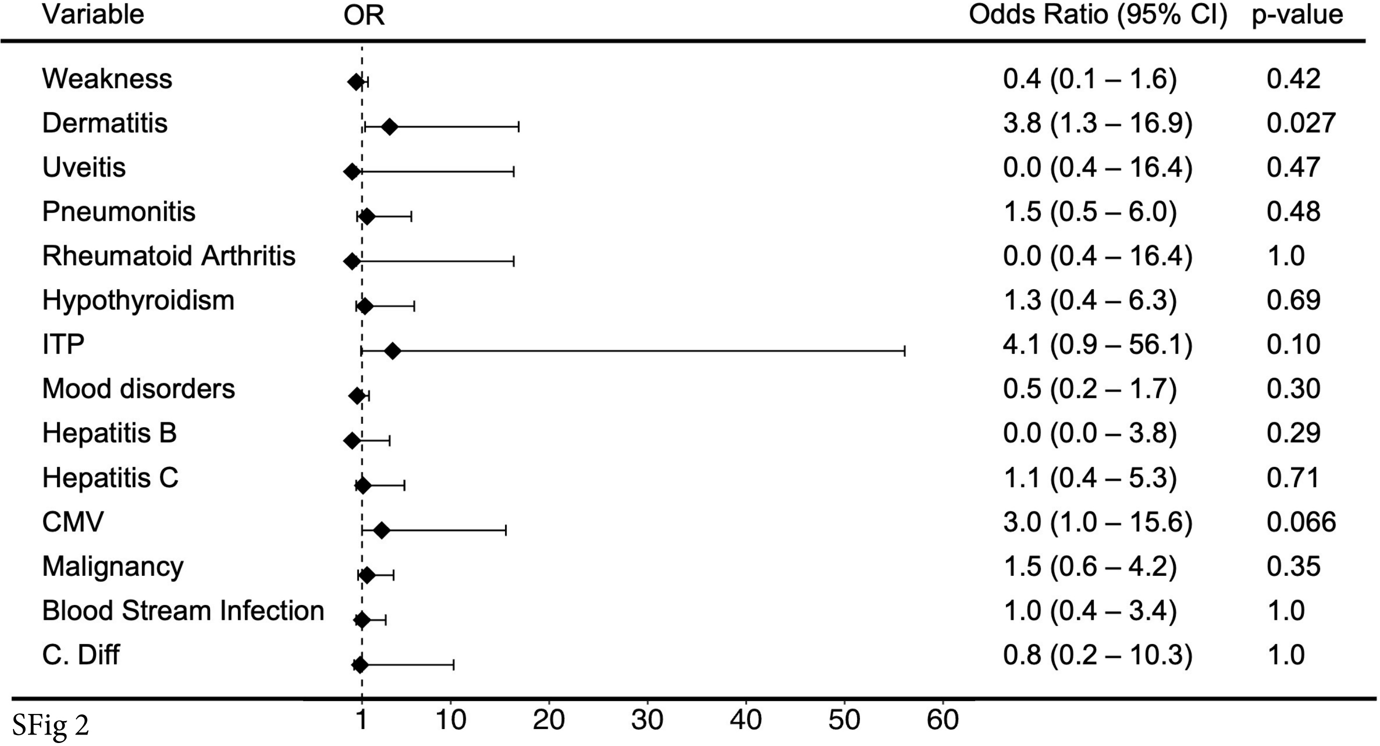

SFig 2

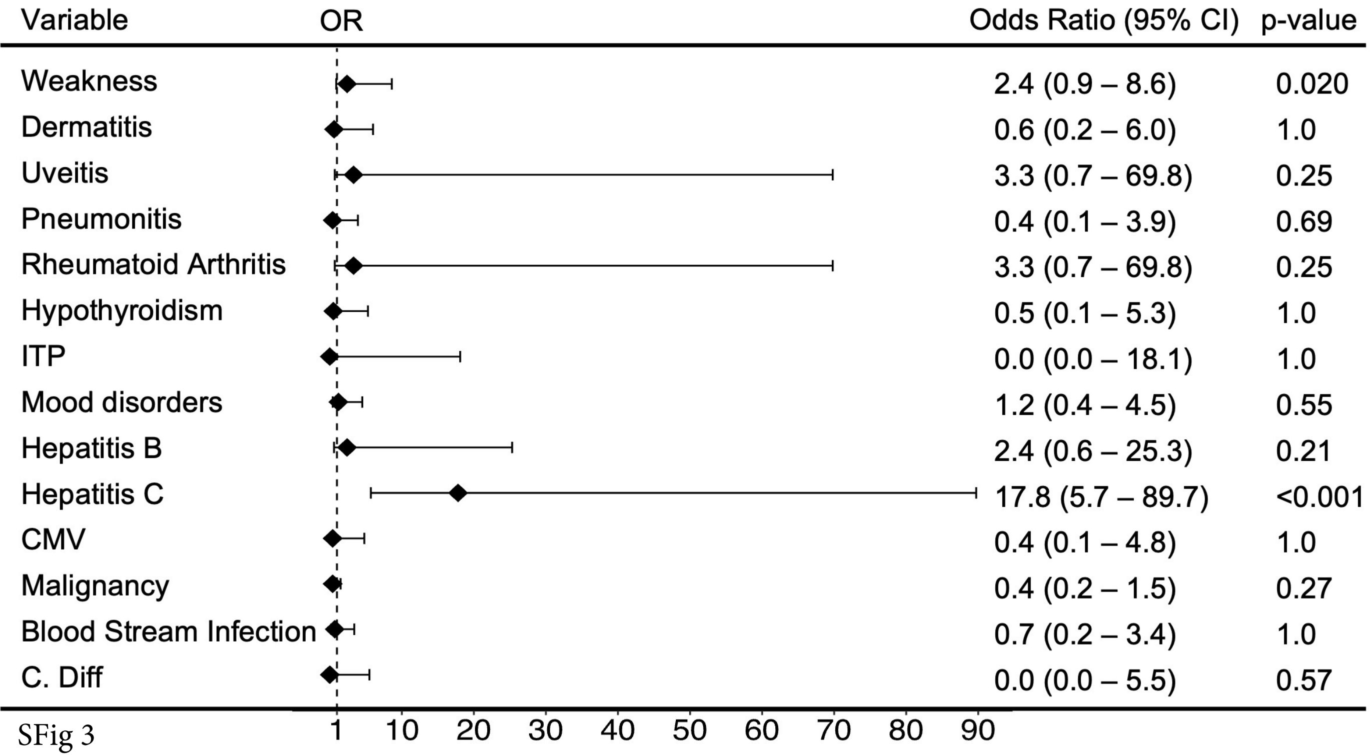
Variable
OR
Odds Ratio (95% CI)
p-value
Weakness 2.4 (0.9 – 8.6) 0.020
Dermatitis 0.6 (0.2 – 6.0) 1.0
Uveitis 3.3 (0.7 – 69.8) 0.25
Pneumonitis 0.4 (0.1 – 3.9) 0.69
Rheumatoid Arthritis 3.3 (0.7 – 69.8) 0.25
Hypothyroidism 0.5 (0.1 – 5.3) 1.0
ITP 0.0 (0.0 – 18.1) 1.0
Mood disorders 1.2 (0.4 – 4.5) 0.55
Hepatitis B 2.4 (0.6 – 25.3) 0.21
Hepatitis C 17.8 (5.7 – 89.7) <0.001
CMV 0.4 (0.1 – 4.8) 1.0
Malignancy 0.4 (0.2 – 1.5) 0.27
Blood Stream Infection 0.7 (0.2 – 3.4) 1.0
C. Diff 0.0 (0.0 – 5.5) 0.57
1 10 20 30 40 50 60 70 80 90

SFig 3

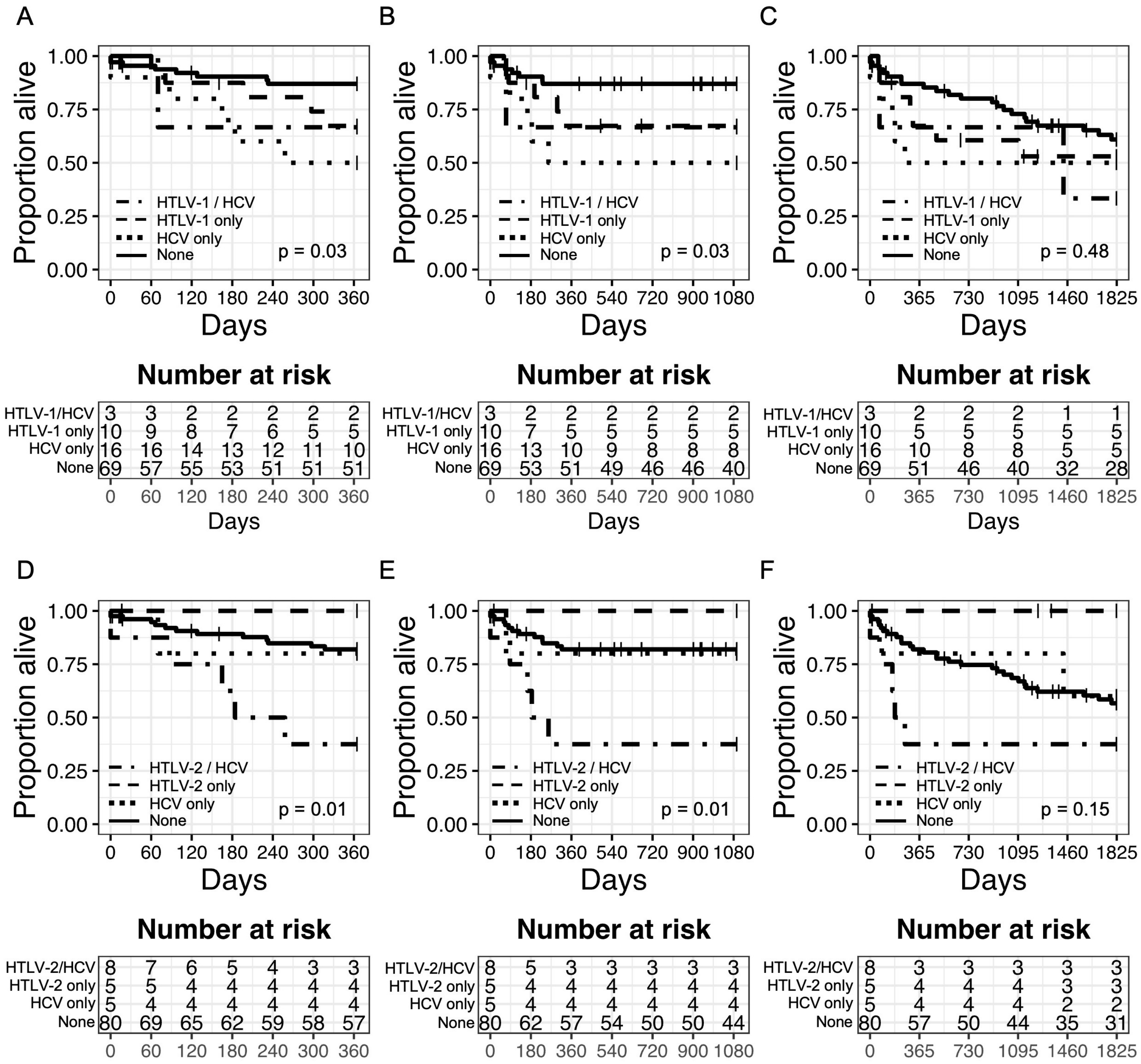
A
Proportion alive
Days
HTLV-1 / HCV
HTLV-1 only
HCV only
None
p = 0.03
Number at risk
HTLV-1/HCV 3 3 2 2 2 2 2
HTLV-1 only 10 9 8 7 6 5 5
HCV only 16 16 14 13 12 11 10
None 69 57 55 53 51 51 51
0 60 120 180 240 300 360
Days
B
Proportion alive
Days
HTLV-1 / HCV
HTLV-1 only
HCV only
None
p = 0.03
Number at risk
HTLV-1/HCV 3 2 2 2 2 2 2
HTLV-1 only 10 7 5 5 5 5 5
HCV only 16 13 10 9 8 8 8
None 69 53 51 49 46 46 40
0 180 360 540 720 900 1080
Days
C
Proportion alive
Days
HTLV-1 / HCV
HTLV-1 only
HCV only
None
p = 0.48
Number at risk
HTLV-1/HCV 3 2 2 2 1 1
HTLV-1 only 10 5 5 5 5 5
HCV only 16 10 8 8 5 5
None 69 51 46 40 32 28
0 365 730 1095 1460 1825
Days
D
Proportion alive
Days
HTLV-2 / HCV
HTLV-2 only
HCV only
None
p = 0.01
Number at risk
HTLV-2/HCV 8 7 6 5 4 3 3
HTLV-2 only 5 5 4 4 4 4 4
HCV only 5 4 4 4 4 4 4
None 80 69 65 62 59 58 57
0 60 120 180 240 300 360
Days
E
Proportion alive
Days
HTLV-2 / HCV
HTLV-2 only
HCV only
None
p = 0.01
Number at risk
HTLV-2/HCV 8 5 3 3 3 3 3
HTLV-2 only 5 4 4 4 4 4 4
HCV only 5 4 4 4 4 4 4
None 80 62 57 54 50 50 44
0 180 360 540 720 900 1080
Days
F
Proportion alive
Days
HTLV-2 / HCV
HTLV-2 only
HCV only
None
p = 0.15
Number at risk
HTLV-2/HCV 8 3 3 3 3 3
HTLV-2 only 5 4 4 4 3 3
HCV only 5 4 4 4 2 2
None 80 57 50 44 35 31
0 365 730 1095 1460 1825
Days

SFig 4

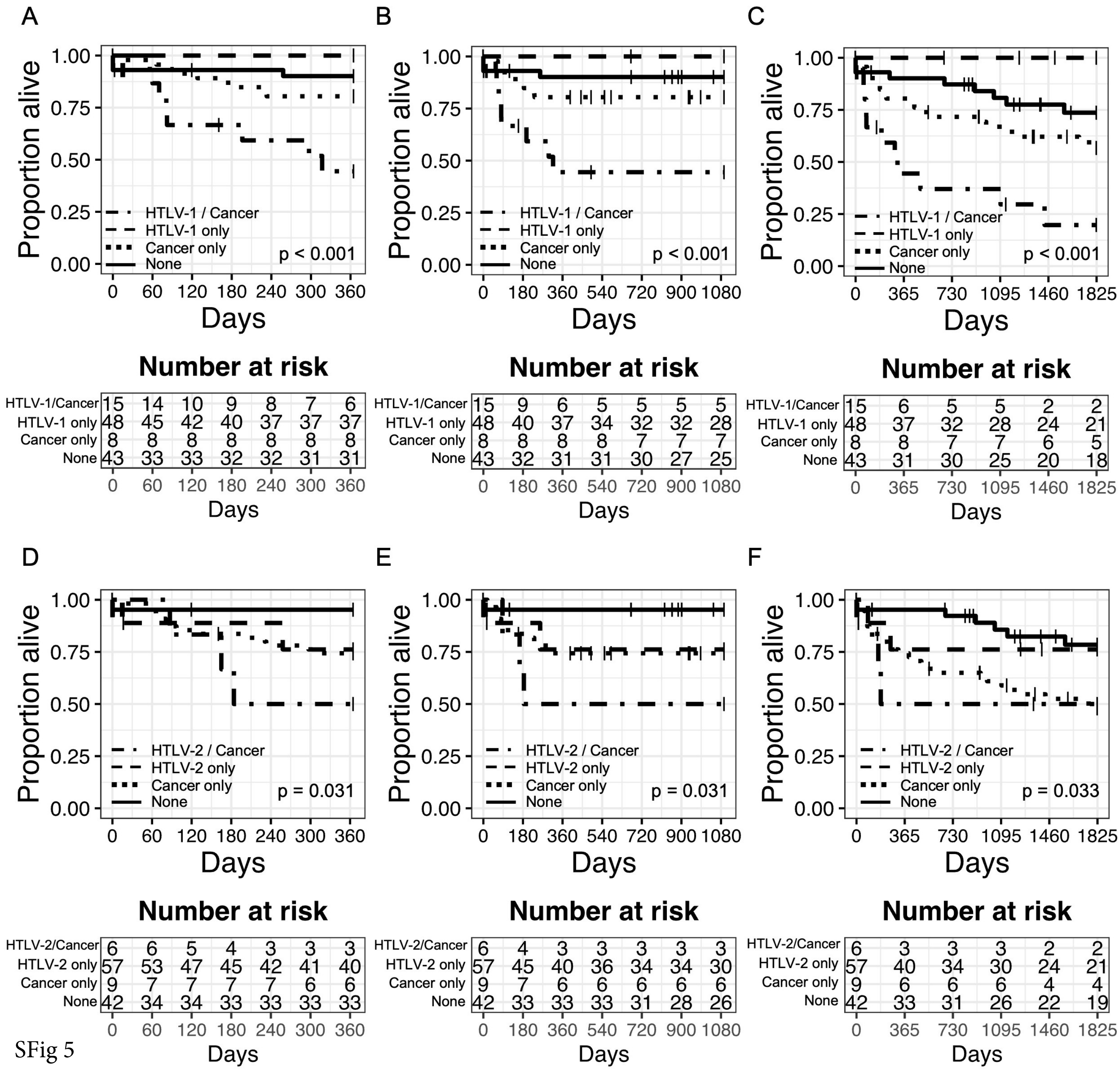
A
Proportion alive
1.00
0.75
0.50
0.25
0.00
HTLV-1 / Cancer
HTLV-1 only
Cancer only
None
p < 0.001
0 60 120 180 240 300 360
Days
Number at risk
HTLV-1/Cancer 15 14 10 9 8 7 6
HTLV-1 only 48 45 42 40 37 37 37
Cancer only 8 8 8 8 8 8 8
None 43 33 33 32 32 31 31
0 60 120 180 240 300 360
Days
B
Proportion alive
1.00
0.75
0.50
0.25
0.00
HTLV-1 / Cancer
HTLV-1 only
Cancer only
None
p < 0.001
0 180 360 540 720 900 1080
Days
Number at risk
HTLV-1/Cancer 15 9 6 5 5 5 5
HTLV-1 only 48 40 37 34 32 32 28
Cancer only 8 8 8 8 7 7 7
None 43 32 31 31 30 27 25
0 180 360 540 720 900 1080
Days
C
Proportion alive
1.00
0.75
0.50
0.25
0.00
HTLV-1 / Cancer
HTLV-1 only
Cancer only
None
p < 0.001
0 365 730 1095 1460 1825
Days
Number at risk
HTLV-1/Cancer 15 6 5 5 2 2
HTLV-1 only 48 37 32 28 24 21
Cancer only 8 8 7 7 6 5
None 43 31 30 25 20 18
0 365 730 1095 1460 1825
Days
D
Proportion alive
1.00
0.75
0.50
0.25
0.00
HTLV-2 / Cancer
HTLV-2 only
Cancer only
None
p = 0.031
0 60 120 180 240 300 360
Days
Number at risk
HTLV-2/Cancer 6 6 5 4 3 3 3
HTLV-2 only 57 53 47 45 42 41 40
Cancer only 9 7 7 7 7 6 6
None 42 34 34 33 33 33 33
0 60 120 180 240 300 360
Days
E
Proportion alive
1.00
0.75
0.50
0.25
0.00
HTLV-2 / Cancer
HTLV-2 only
Cancer only
None
p = 0.031
0 180 360 540 720 900 1080
Days
Number at risk
HTLV-2/Cancer 6 4 3 3 3 3 3
HTLV-2 only 57 45 40 36 34 34 30
Cancer only 9 7 6 6 6 6 6
None 42 33 33 33 31 28 26
0 180 360 540 720 900 1080
Days
F
Proportion alive
1.00
0.75
0.50
0.25
0.00
HTLV-2 / Cancer
HTLV-2 only
Cancer only
None
p = 0.033
0 365 730 1095 1460 1825
Days
Number at risk
HTLV-2/Cancer 6 3 3 3 2 2
HTLV-2 only 57 40 34 30 24 21
Cancer only 9 6 6 6 4 4
None 42 33 31 26 22 19
0 365 730 1095 1460 1825
Days

SFig 5

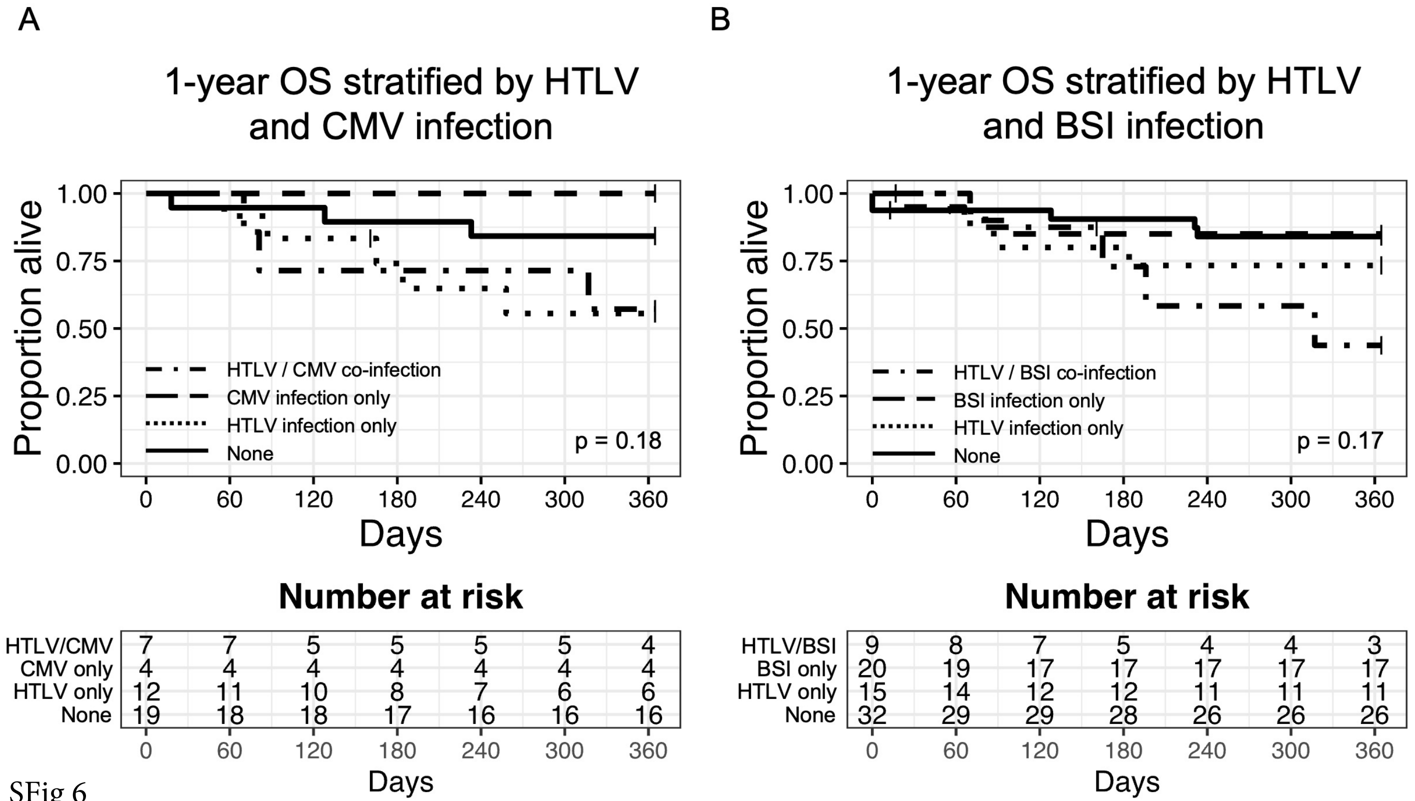
A
1-year OS stratified by HTLV and CMV infection
Proportion alive
1.00
0.75
0.50
0.25
0.00
HTLV / CMV co-infection
CMV infection only
HTLV infection only
None
p = 0.18
0 60 120 180 240 300 360
Days
Number at risk
HTLV/CMV 7 7 5 5 5 5 4
CMV only 4 4 4 4 4 4 4
HTLV only 12 11 10 8 7 6 6
None 19 18 18 17 16 16 16
0 60 120 180 240 300 360
Days
B
1-year OS stratified by HTLV and BSI infection
Proportion alive
1.00
0.75
0.50
0.25
0.00
HTLV / BSI co-infection
BSI infection only
HTLV infection only
None
p = 0.17
0 60 120 180 240 300 360
Days
Number at risk
HTLV/BSI 9 8 7 5 4 4 3
BSI only 20 19 17 17 17 17 17
HTLV only 15 14 12 12 11 11 11
None 32 29 29 28 26 26 26
0 60 120 180 240 300 360
Days

SFig 6
